# BTG1 expression correlates with pathogenesis, aggressive behaviors and prognosis of gastric cancer: a potential target for gene therapy

**DOI:** 10.18632/oncotarget.4081

**Published:** 2015-06-05

**Authors:** Hua-chuan Zheng, Jing Li, Dao-fu Shen, Xue-feng Yang, Shuang Zhao, Ya-zhou Wu, Yasuo Takano, Hong-zhi Sun, Rong-jian Su, Jun-sheng Luo, Wen-feng Gou

**Affiliations:** ^1^ Cancer Research Center, Key Laboratory of Brain and Spinal Cord Injury of Liaoning Province, and Laboratory Animal Center, The First Affiliated Hospital of Liaoning Medical University, Jinzhou, China; ^2^ Department of Gastroenterology, The First Affiliated Hospital of Liaoning Medical University, Jinzhou, China; ^3^ School of Health Science, Tokyo University of Technology, Ohta-ku, Tokyo, Japan; ^4^ Experimental Center, Liaoning Medical University, Jinzhou, China

**Keywords:** gastric cancer, BTG1, pathobiological behaviors, carcinogenesis, gene therapy

## Abstract

Here, we found that BTG1 overexpression inhibited proliferation, migration and invasion, induced G_2_/M arrest, differentiation, senescence and apoptosis in BGC-823 and MKN28 cells (*p* < 0.05). *BTG1* transfectants showed a higher mRNA expression of *Cyclin D1* and *Bax*, but a lower mRNA expression of *cdc2, p21, mTOR* and *MMP-9* than the control and mock (*p* < 0.05). After treated with cisplatin, MG132, paclitaxel and SAHA, both *BTG1* transfectants showed lower mRNA viability and higher apoptosis than the control in both time- and dose-dependent manners (*p* < 0.05) with the hypoexpression of chemoresistance-related genes (*slug*, *CD147*, *GRP78*, *GRP94*, *FBXW7 TOP1*, *TOP2* and *GST-π*). *BTG1* expression was restored after 5-aza-2′-deoxycytidine treatment in gastric cancer cells. BTG1 expression was statistically lower in gastric cancer than non-neoplastic mucosa and metastatic cancer in lymph node (*p* < 0.05). BTG1 expression was positively correlated with depth of invasion, lymphatic and venous invasion, lymph node metastasis, TNM staging and worse prognosis (*p* < 0.05). The diffuse-type carcinoma showed less BTG1 expression than intestinal- and mixed-type ones (*p* < 0.05). BTG1 overexpression suppressed tumor growth and lung metastasis of gastric cancer cells by inhibiting proliferation, enhancing autophagy and apoptosis in xenograft models. It was suggested that down-regulated *BTG1* expression might promote gastric carcinogenesis partially due to its promoter methylation. BTG1 overexpression might reverse the aggressive phenotypes and be employed as a potential target for gene therapy of gastric cancer.

## INTRODUCTION

Despite a worldwide decline in incidence and mortality since the second half of the 20th century, gastric cancer remains the fourth most common and the second most frequent death cause from cancer [1, 2]. Although the patients with gastric cancer are treated with surgery, chemotherapy, radiotherapy, immunotherapy or gene therapy, the identification of novel biomarkers and gene therapy targets for cancer diagnosis and treatment may result in the improvement of survival time and quality of the cancer patients.

BTG (B-cell translocation gene) family comprises six proteins (BTG1, BTG2, BTG3, BTG4, Transducer of ErbB-2, and TOB2), which inhibit cell proliferation and cell cycle progression, induce differentiation in various cells. BTG proteins can shuttle in nucleocytoplasmic counterparts because of their nuclear localization and export signals [3]. There is a highest BTG1 expression in G_0_/G_1_ phases and its down-regulation with cells cycle progressing through G_1_ phase [4]. Further investigation shows that BTG1 protein can bind to nuclear receptor TRα and the myogenic factor MyoD [5], protein arginine methyltransferase 1 [6], and human carbon catabolite repressor protein-associative factor 1 [7]. BTG1 overexpression was detectable in apoptotic cells [8] and helpful for anti-sense Bcl-2-induced cytotoxic effects [9]. BTG1 was reported to enhance Hoxb9-induced transcription to suppress proliferation in HeLa cells [10]. In agreement with the report of Zhu et al. [11], we found that BTG1 overexpression suppressed proliferation, migration and invasion, and induced chemosensitivity to cisplatin, G_1_ arrest and apoptosis of ovarian cancer cells [12]. There was a higher expression of *BTG1* mRNA in normal tissue than in cancer tissue, and in benign tumors than in cancer tissue of the ovary. *BTG1* mRNA expression was inversely related to FIGO staging of ovarian cancers [12].

To clarify the roles of *BTG1* in gastric carcinogenesis and subsequent progression, we examined the expression of *BTG1* mRNA and protein, and its promoter methylation in gastric cancer tissues, and compared them with the clinicopathological and prognostic parameters of the cancers. In addition, we focused on the effects of BTG1 overexpression on the aggressive phenotypes of gastric cancer cells and clarified the relevant molecular mechanisms.

## RESULTS

### The expression and promoter methylation of *BTG1* in gastric cancer cells

**Figure 1:**
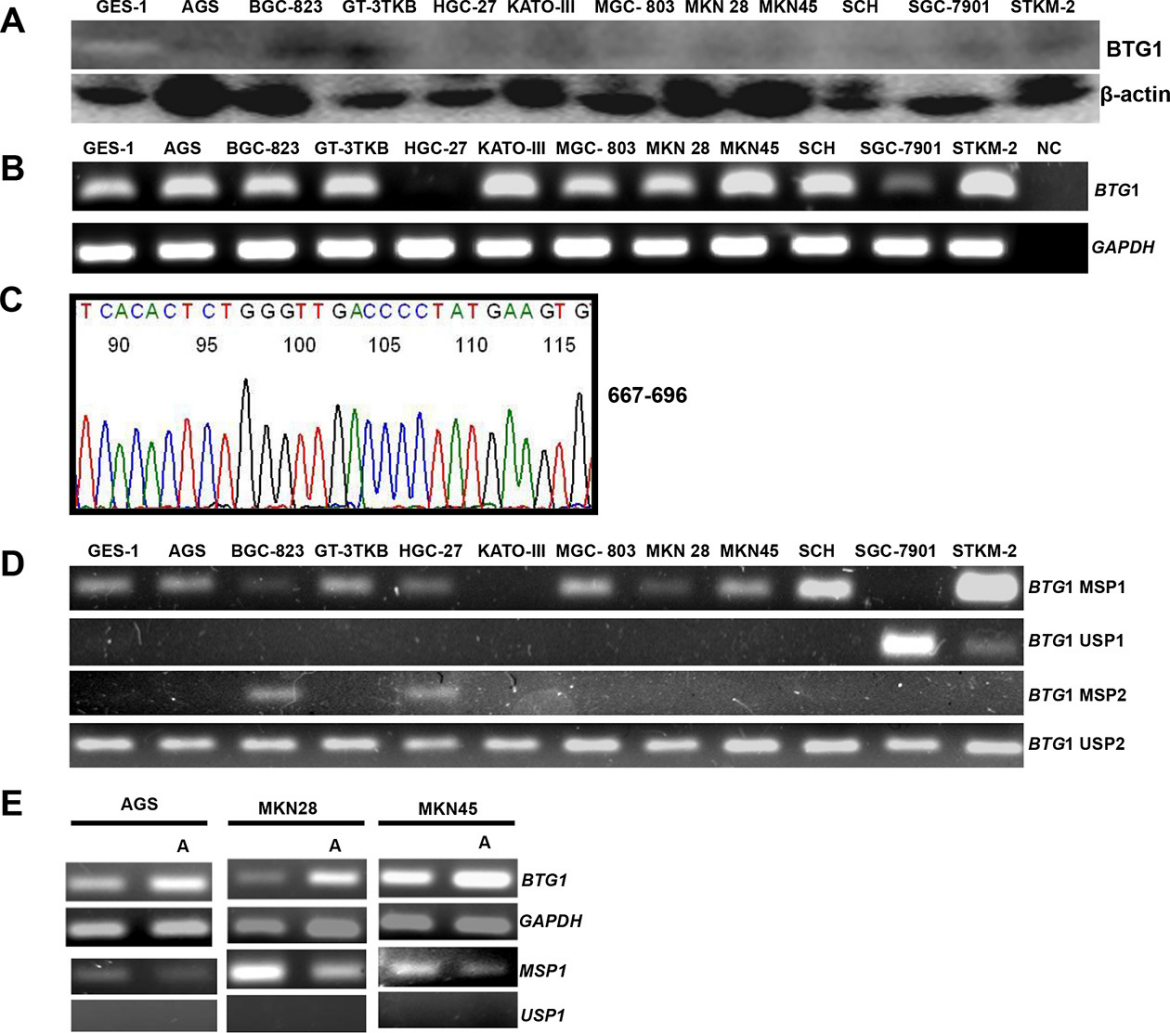
BTG1 expression and methylation in gastric cancer cell lines BTG1 protein expression (19 kDa) was detectable in gastric cancer and epithelial cell lines at a very weak level with β-actin (42 kDa) as an internal control **A.** *BTG1* expression was strong in cancer and epithelial cells by RT-PCR, except HCG-27 and SGC-7901 **B.** followed by direct sequencing **C.** *BTG1* methylation was found in gastric cancer or epithelial cell lines **D.** AGS, MKN28 and MKN45 cells showed a high expression of *BTG1* mRNA by RT-PCR and a decreased level of methylation of *BTG1* after treated with 5-Aza-dC **E.** Note: NC, negative control; U, unmethylated; M, methylated; MSP, methylation-specific PCR; A, 5′-Aza-2′-deoxycytidine.

BTG1 protein was expressed in gastric cancer or epithelial cells at different levels by Western blot (Figure 1A). To check its mRNA expression, we employed RT-PCR and observed strong expression of *BTG1* mRNA in GES-1, AGS, BGC-823, GT-3 TKB, KATO-III, MGC-803, MKN28, MKN45, SCH, and STKM-2, but not or weakly in HGC-27 or SGC-7901 (Figure 1B). The amplicons proved correct by direct DNA sequencing (Figure 1C). We detected *BTG1* promoter methylation in GES-1, AGS, BGC-823, GT-3 TKB, HGC-27, MGC-803, MKN28, MKN45, SCH, and STKM-2 using methylation-specific PCR, but not or weakly in KATO-III or SGC-7901 (Figure 1D). After treatment with demethylation reagent (5-Aza-dC), we found that *BTG1* promoter methylation was decreased and its mRNA expression was restored in AGS, MKN28 and MKN45 cells (Figure 1E).

### The effects of BTG1 overexpression on biological phenotypes of gastric cancer cells

**Figure 2:**
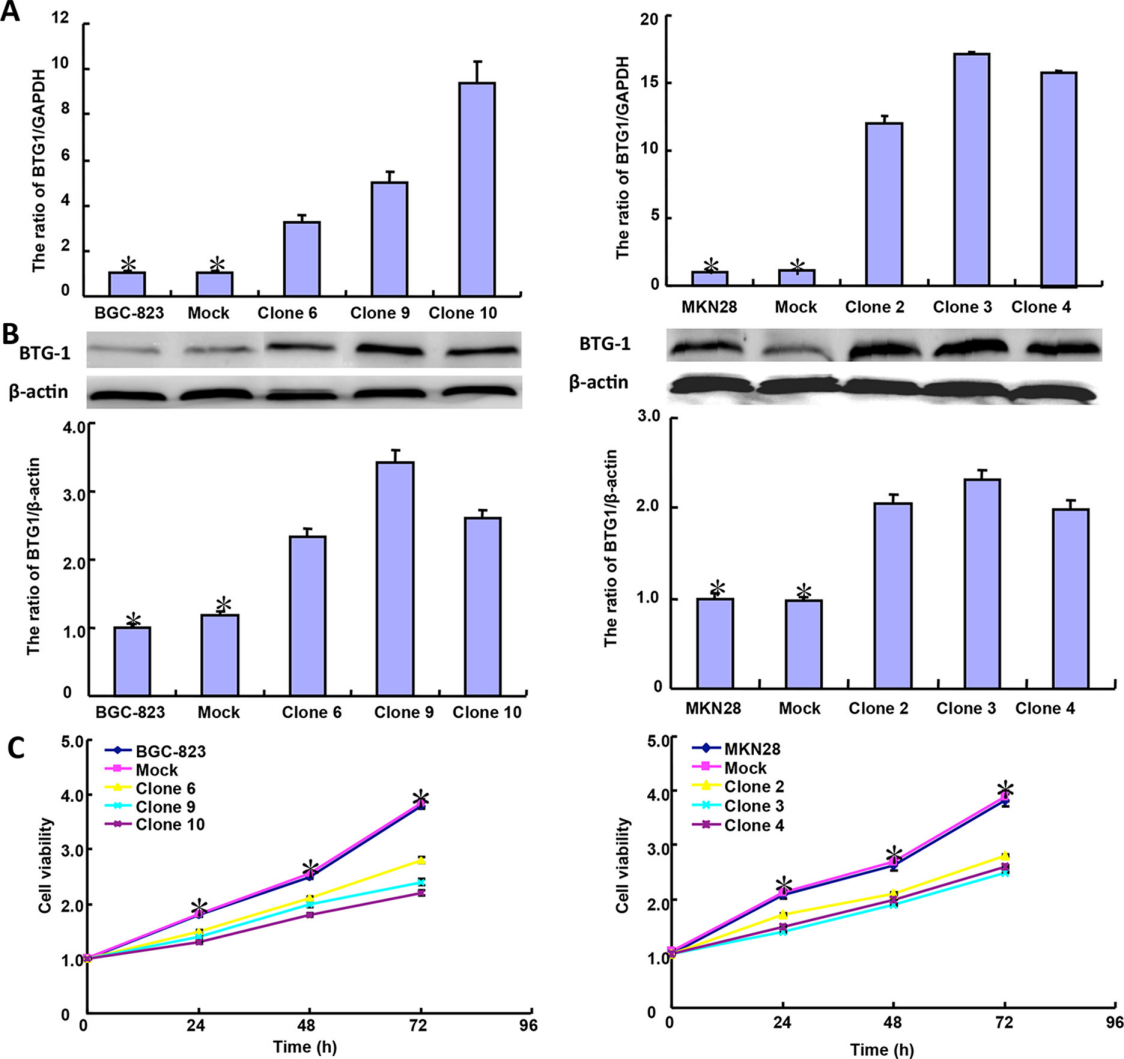

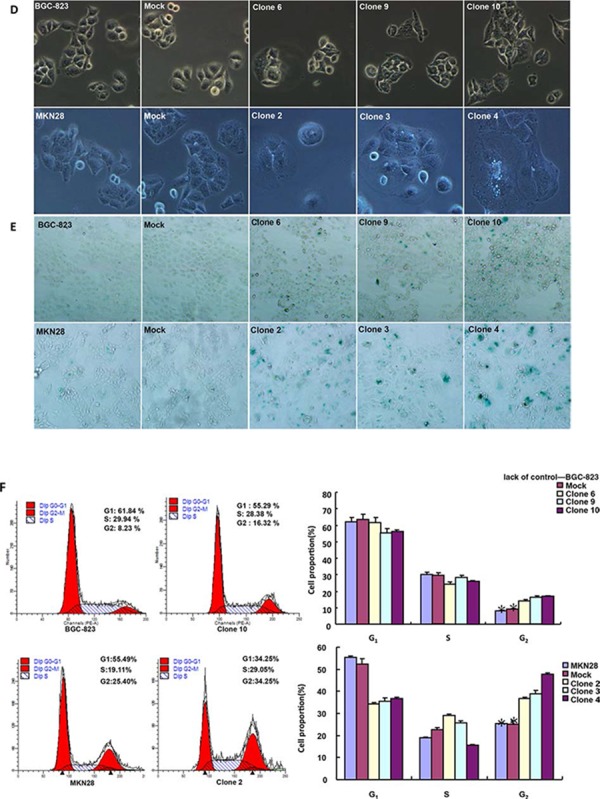

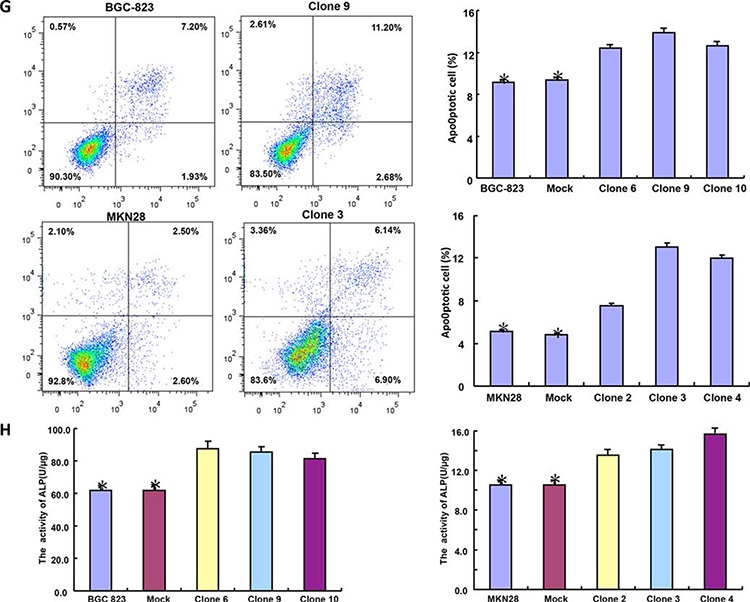

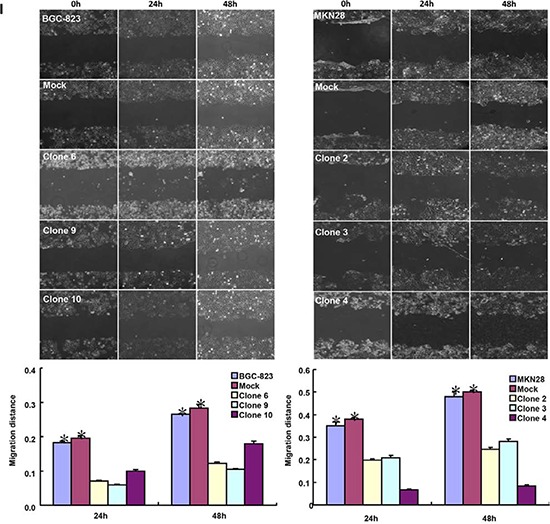

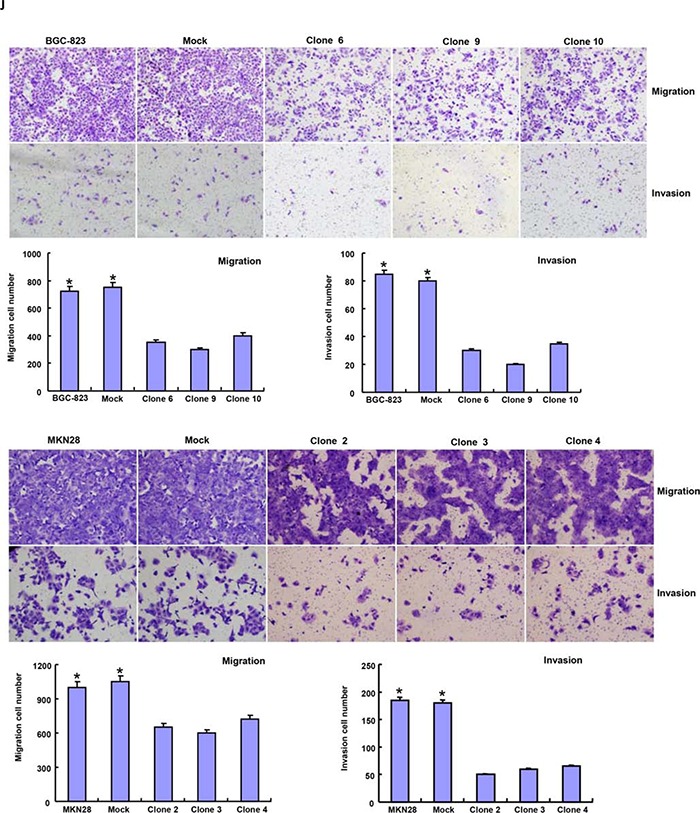

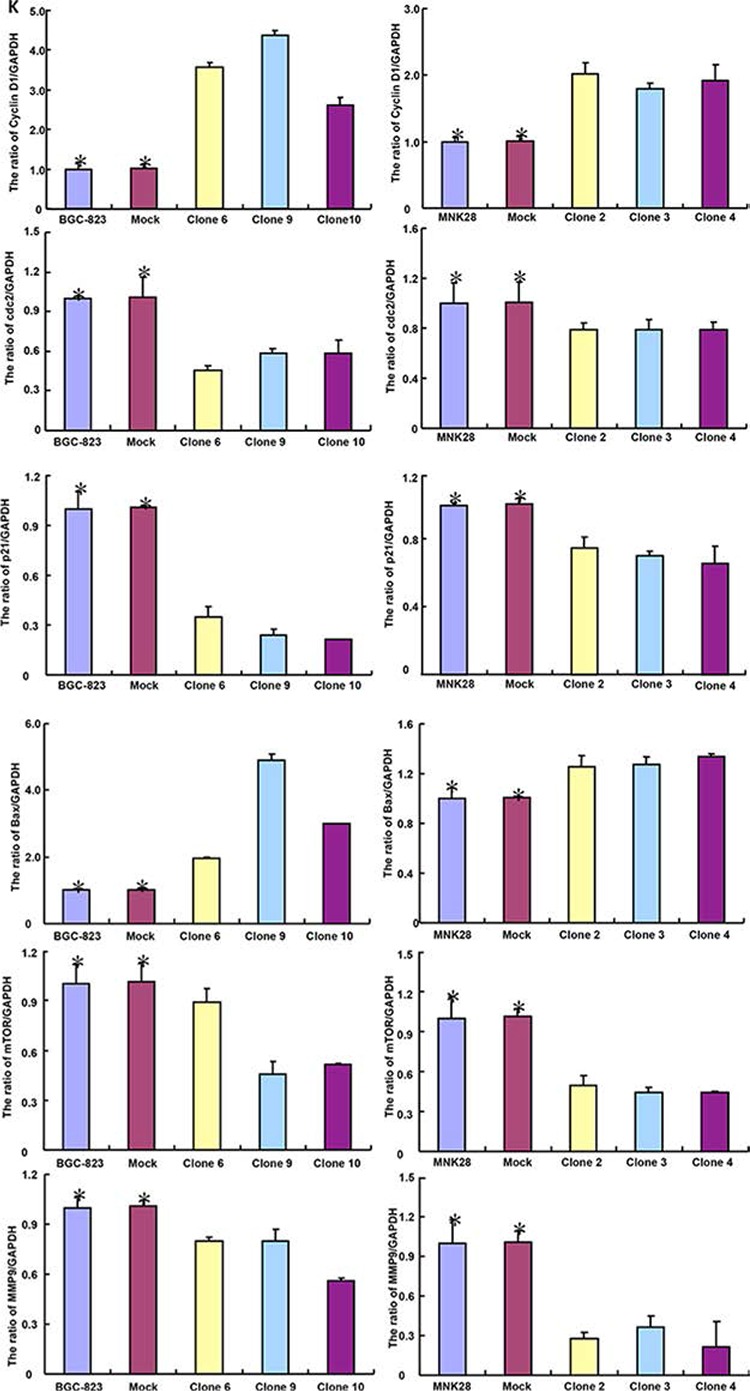
BTG1 overexpression represses the aggressive phenotypes of gastric cancer cells and causes the alteration in the related gene expression After transfection of pcDNA3.1-*BTG1*, BTG1 expression became strong in BGC-823 and MKN28 cells by RT-PCR **A.** and Western blot **B.** The transfectants showed a lower growth **C.** and mitotic dysfunction. **D.** and a higher senescence **E.** than the control and mock. PI staining showed G_2_/M arrest in *BTG1* transfectants **F.** There was both apoptosis- and differentiation-induced effect of BTG1 overexpression on gastric cancer cells, evidenced by Annexin V assay **G.** and alkaline phosphatase (ALP) activity **H.** *BTG1*-overexpressing cells had a weaker ability to migrate and invade than the control and mock according to the results of wound healing **I.** and transwell chamber assay **J.** The expression of phenotype-related molecules was screened by real-time RT-PCR **K.** *p < 0.05, compared with the transfectants.

After transfected with pcDNA3.1-*BTG1*, both BGC-823 and MKN28 cells overexpressed *BTG1* at both protein and mRNA levels (Figure 2A and 2B). There was slower growth (Figure 2C, *p* < 0.05), mitotic disruption (Figure 2D) and higher senescence (Figure 2E) in *BTG1* transfectants than the control and mock cells. Cell cycle analysis indicated that G_2_/M arrest in *BTG1* transfectants (Figure 2F). There was a higher level of apoptosis evidenced by Annexin-V (Figure 2G, *p* < 0.05) and a better differentiation by ALP activity (Figure 2H, *p* < 0.05) in *BTG1* transfectants than the control and mock. Additionally, BTG1 overexpression could suppress migration and invasion by wound healing (Figure 2I, *p* < 0.05) or transwell chamber assay (Figure 2J, *p* < 0.05). *BTG1* transfectants showed a higher expression of *Cyclin D1 and Bax,* but a lower expression of *cdc2, p21, mTOR and MMP-9* than the control and mock (Figure 2K, *p* < 0.05).

**Figure 3:**
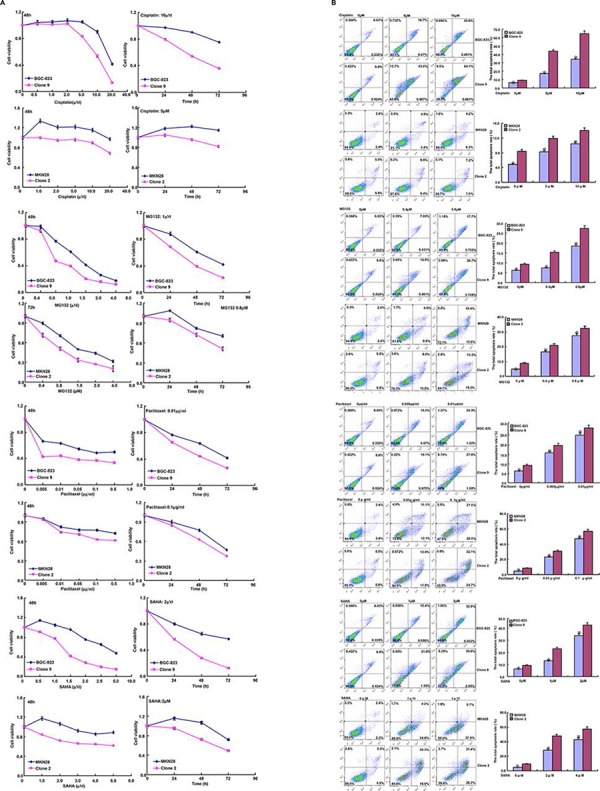

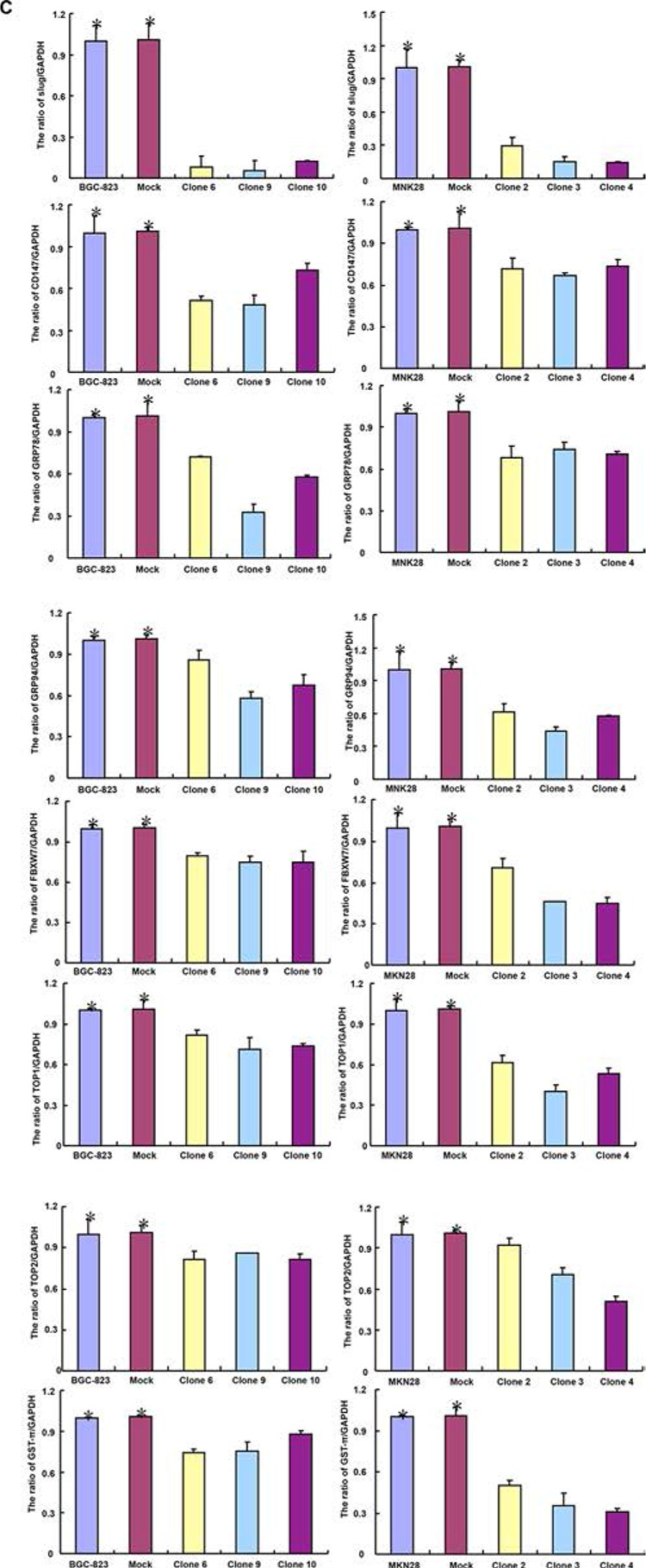
BTG1 expression enhances the sensitivity of gastric cancer cells to chemotherapeutic agents Exposed to cisplatin, MG132, paclitaxel and SAHA, *BTG1* transfectants showed a lower viability and a higher apoptotic level than the control at both concentration- and time-dependent manners **A and B.** The chemoresistance-related genes were screened by real-time RT-PCR **C.** *p < 0.05, compared with the transfectant.

After treated with cisplatin, MG132, paclitaxel and SAHA, both *BTG1* transfectants displayed lower viability and higher apoptosis than the control in both time- and dose-dependent manners (Figure 3A–3B, *p* < 0.05). BTG1 overexpression decreased the expression of *slug*, *CD147*, *GRP78*, *GRP94*, *FBXW7*, *TOP1*, *TOP2*, and *GST-π* in BGC-823 and MKN28 cells (Figure 3C, *p* < 0.05).

### The relationship between BTG1 expression and clinicopathological or prognostic parameters of gastric cancers

**Figure 4:**
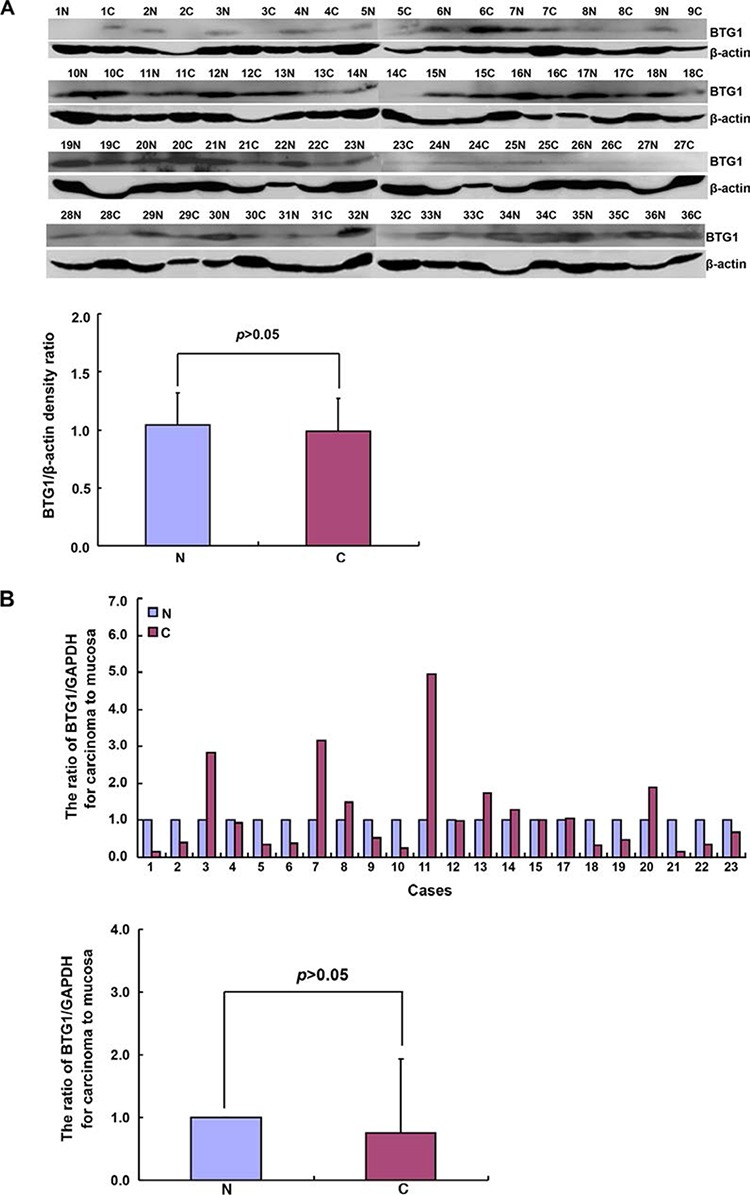

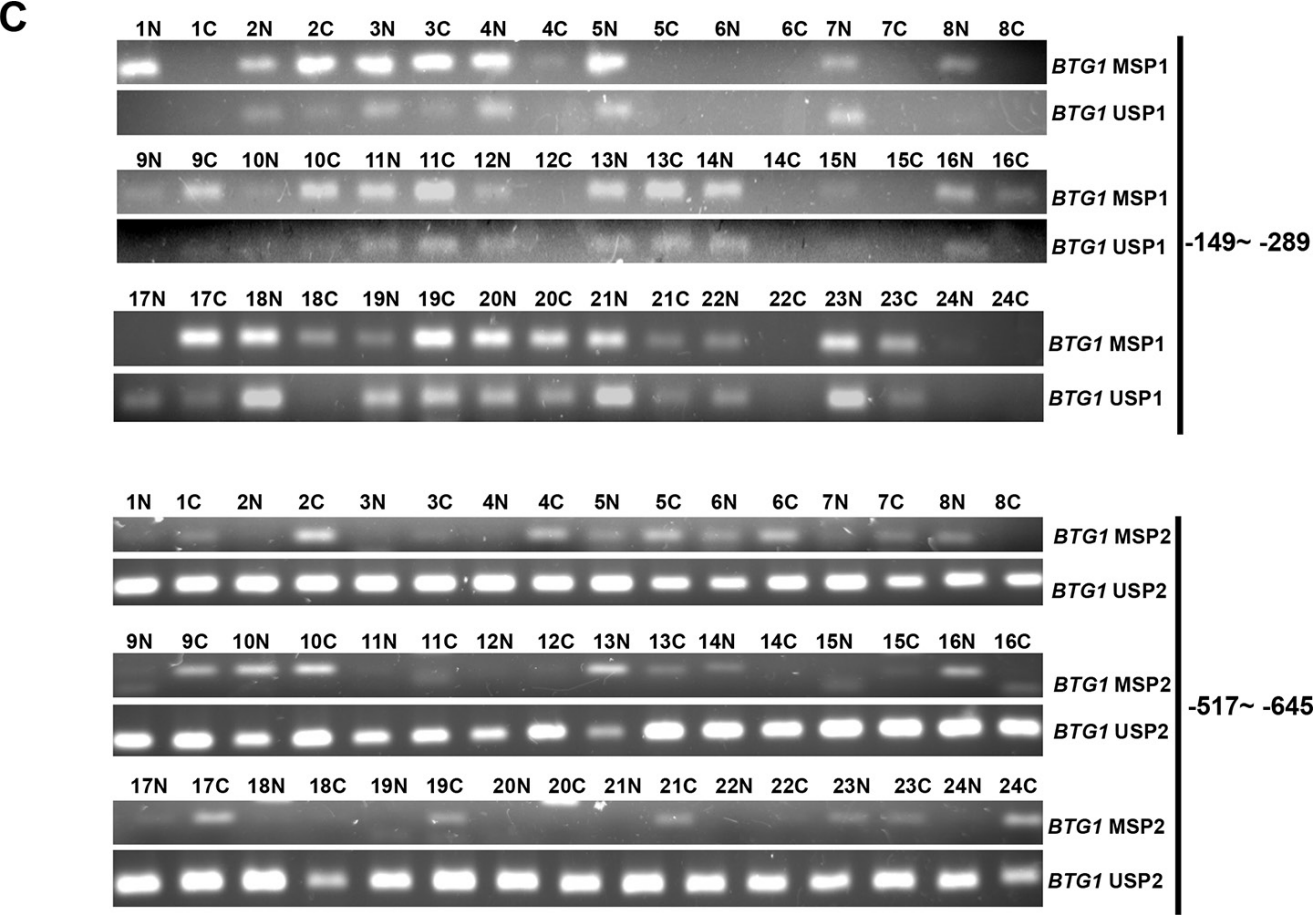
BTG1 expression and methylation in gastric cancer and matched mucosa According to Western blot, densitometry analysis showed no difference in BTG1 protein expression (19 kDa) between gastric cancer and matched mucosa with β-actin (42 kDa) as an internal control (*p* > 0.05, **A**). *BTG1* was amplified by real-time RT-PCR with *GAPDH* as an internal control and no difference in *BTG1* mRNA expression was found between cancer than paired mucosa (*p* > 0.05, **B**). Methylation-specific PCR was performed using gastric cancer and matched mucosa tissues **C**. Note: M, methylated; U, unmethylated; N, non-neoplastic mucosa; C, cancer; NC, negative control.

BTG1 protein was detected in gastric cancer and adjacent non-neoplastic mucosa (NNM) at an approximately equal level (*p* > 0.05, Figure 4A). Compared with NNM, decreased *BTG1* mRNA expression was seen in 57.4% (13/23) of gastric cancers, while there was no statistical difference between gastric cancer and paired NNM (Figure 4B). Although we designed methylation-specific primers of different *BTG1* promoter regions, no difference in *BTG1* promoter methylation was found between gastric cancer and NNM (Figure 4C; 58.3% *vs* 83.3%; 66.7% *vs* 45.8%; *p* > 0.05). *BTG1* methylation was not correlated with its mRNA expression in gastric cancer or NNM (*p* > 0.05, data not shown)

**Figure 5:**
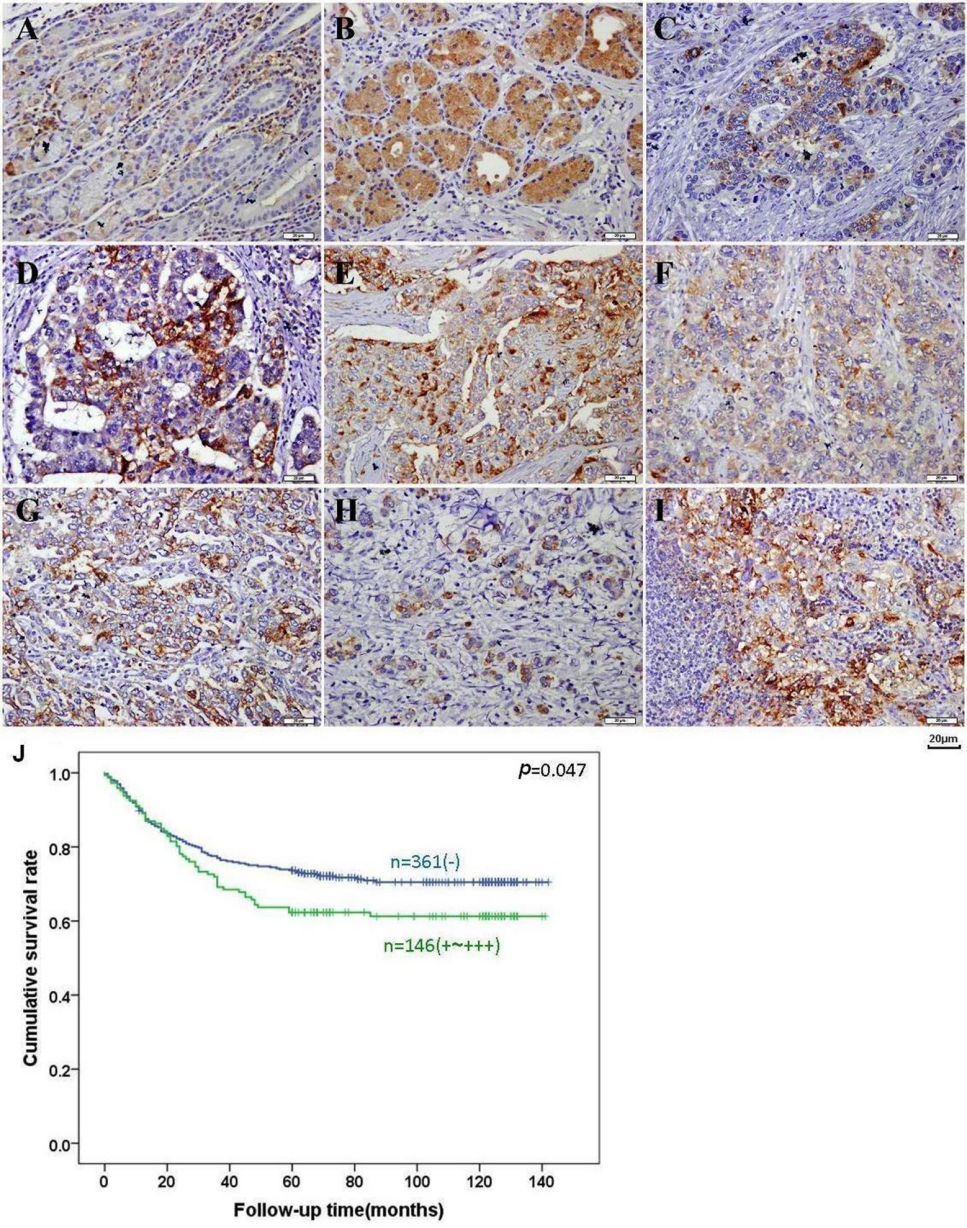
BTG1 expression and prognostic significance in gastric cancer BTG1 protein was positively detected in the cytoplasm of the superficial epithelia cells, infiltrating inflammatory cells **A.** deep propria glands **B.** well-**C.** moderately- **D.** poorly- **F-G.** differentiated, signet ring cell **H.** carcinoma and metastatic cancer in lymph node **I.** *Kaplan-Meier* analysis showed negative correlation between BTG1 expression and cumulative survival rate of patients with gastric cancer **J.** Note: the bar indicates 20 μm.

As shown in Figure 5A–5I, BTG1 protein was distributed in the cytoplasm of infiltrating inflammatory cells, deep propria glands, fundic glands, well-, moderately-, poorly-differentiated, and signet ring cell carcinomas, and metastatic cancer in lymph node, occasionally in the superficial mucosa. BTG1 expression was detectable in gastric NNM (52.9%, 305/577), primary cancers (27.7%, 170/613), and metastatic cancers in lymph node (48.6%, 86/177), respectively. According to its frequency and density, BTG1 expression was statistically lower in primary cancers than adjacent NNM and metastatic cancers in lymph node (*p* < 0.05, Table 1).

**Table 1:** BTG1 expression in gastric non-neoplastic mucosa, primary and metastatic cancers

Groups	n	BTG1 expression				
		−	+	++	+++	PR(%)
Non-neoplastic mucosa	577	272	86	102	117	52.9
Primary cancer	613	443	90	54	26	27.7*
Metastatic cancer in lymph node	177	91	49	21	16	48.6

* compared with non-neoplastic mucosa or metastatic cancer, *p* < 0.001.

PR = positive rate

As summarized in Table 2, BTG1 was more expressed in elder male cancer patients than that in their younger female counterparts (*p* < 0.05). BTG1 expression was positively correlated with depth of invasion, lymphatic and venous invasion, lymph node metastasis, and TNM staging (*p* < 0.05), but not with distant metastasis (*p* > 0.05). The diffuse-type carcinomas showed less BTG1 expression than intestinal- and mixed-type ones (*p* < 0.05). There was no difference in BTG1 expression between intestinal and diffuse components of mixed-type carcinomas (*p* > 0.05, data not shown). It was the same between primary and matched metastatic cancers (*p* > 0.05, data not shown).

**Table 2:** Relationship between BTG1 expression and clinicopathological features of gastric cancers

Clinicopathological features	n	BTG1 expression					
		−	+	++	+++	PR(%)	*p* value
Age(year)							0.003
< 65	351	270	43	28	10	23.1	
≥ 65	262	173	47	26	16	34.0	
Sex							0.034
Male	427	297	65	45	20	30.4	
Female	186	146	25	9	6	21.5	
Depth of invasion							< 0.001
T_is-1_	294	239	25	22	8	18.7	
T_2-4_	310	197	63	32	18	36.5	
Lymphatic invasion							< 0.001
−	354	286	38	20	10	19.2	
+	259	157	52	34	16	39.4	
Venous invasion							< 0.001
−	346	285	36	18	7	17.6	
+	267	158	54	36	19	40.8	
Lymph node metastasis							0.001
−	348	270	40	29	9	22.4	
+	265	173	50	25	17	34.7	
Distant metastasis							0.812
−	578	418	83	52	25	27.7	
+	35	25	7	2	1	28.6	
TNM staging							0.001
I-II	355	276	38	31	10	22.3	
III-IV	244	158	47	23	16	35.2	
Lauren's classification							—
Intestinal-type	186	129	32	18	7	30.6	
Diffuse-type	219	186	16	10	7	15.1^*^	
Mixed-type	204	126	41	26	11	38.2	

PR, positive rate; T_is_ = cancer in situ; T_1_ = lamina propria and submucosa; T_2_ = muscularis propria and subserosa; T_3_ = exposure to serosa; T_4_ = invasion into serosa; TNM = tumor-node-metastasis

* compared with intestinal-type or mixed-type carcinoma, *p* < 0.001.

Follow-up information was available on 507 cancer patients for periods ranging from 2 days to 10.8 years (median = 69 months). Univariate analysis using Kaplan-Meier method indicated that the cumulative survival rate of the cancer patients with weak, moderate, or strong expression of BTG1 was obviously lower than that without its expression (Figure 5J, *p* < 0.05). If stratified according to depth of invasion, this significant relationship disappeared (data not shown). Multivariate analysis using Cox's proportional hazard model showed that venous invasion, distant metastasis and TNM staging (*p* < 0.05), but not age, sex, depth of invasion, lymphatic invasion, lymph node metastasis, Lauren's classification or BTG1 expression (*p* > 0.05) were independent prognostic factors for overall gastric cancer patients (Table 3).

**Table 3:** Multivariate analysis of clinicopathological variables for survival of gastric cancer patients

Clinicopathological parameters	Relative risk (95%CI)	*p* value
Age (≥ 65years)	1.380(0.998 – 1.909)	0.052
Sex (female)	0.703(0.484 – 1.020)	0.064
Depth of invasion (T_2–4_)	1.863(0.633 – 5.490)	0.259
Lymphatic invasion (+)	1.491(0.955 – 2.329)	0.079
Venous invasion (+)	1.660(1.017 – 2.707)	0.042
Lymph node metastasis (+)	1.842(0.993 – 3.418)	0.053
Distant metastasis (+)	4.168(2.649 – 6.558)	< 0.001
TNM staging (III–IV)	5.586(2.529 – 12.339)	< 0.001
Lauren's classification (IT/DT/MT)	1.129(0.897 – 1.420)	0.301
BTG1 expression (+∼+++)	0.813(0.578 – 1.143)	0.233

CI, confidence interval; TNM, tumor-node-metastasis; IT, intestinal-type; DT, diffuse-type; MT, mixed-type.

### BTG1 suppresses the growth and lung metastasis of gastric cancer cells

**Figure 6:**
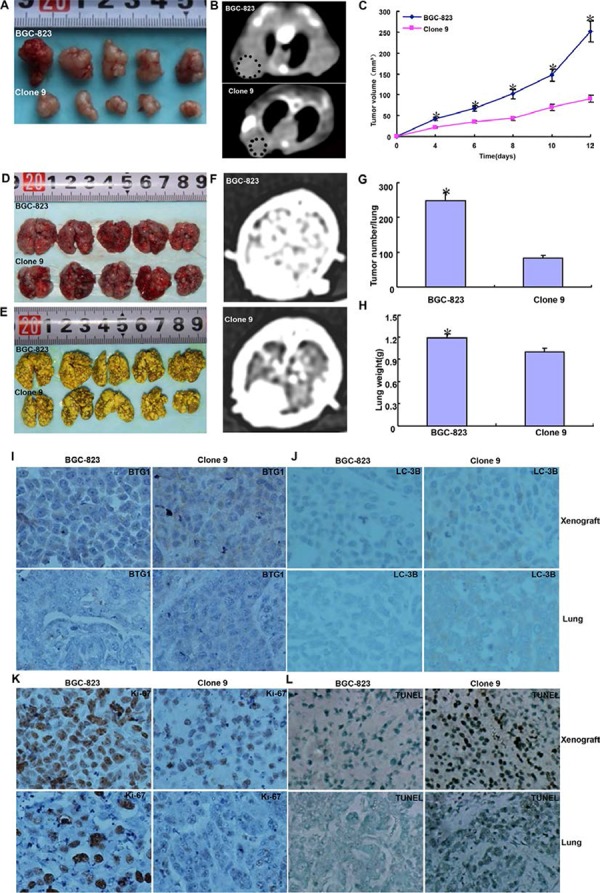
BTG1 suppresses the growth and lung metastasis of gastric cancer cells The growth of BGC-823 cells was faster than their BTG1 tranfectants by gross appearance **A.** CT scanning **B.** and measuring tumor volume (**C**, *p* < 0.05). BGC-823 cells showed more lung metastasis foci and heavier lung weight than their *BTG1* transfectants by gross observation **D.** mandelic acid staining **E.** contrast CT scanning **F.** number counting (**G**, *p* < 0.05) and weighting (H, *p* < 0.05). The transfectant cells showed stronger expression of BTG1 **I.** ki-67 **J.** and LC-3B **K.** than the control in subcutaneous tumor and lung metastatic foci. The apoptotic level was higher in BTG1 tranfectants than maternal cells by TUNEL assay **L.** *p < 0.05, compared with the transfectant.

BGC-823 cells and *BTG1* transfectants were subcutaneously and intravenously transplanted into the axilla and tail vein of nude mice. The tumor volume of BGC-823 cells xenograft was larger than that of *BTG1* transfectants by gross appearance, CT scanning and ruling respectively (Figure 6A–6C, *p* < 0.05). It was the same for tumor number of lung metastasis by gross appearance, contrast CT scanning and counting (Figure 6D–6G, *p* < 0.05). The lung weight of *BTG1* transfectants appeared lower than the control in lung metastasis model (Figure 6H, *p* < 0.05). Immunohistochemical data showed that BGC-823 transfectants showed higher BTG1 expression, lower proliferation by ki-67 marker, higher authophagy by LC-3B staining, and stronger apoptosis by TUNEL assay than the control (Figure 6I–6L). The same results were obtained in MKN28 (data not shown).

## DISCUSSION

Here, we found that BTG1 protein was mainly localized in the cytoplasm of superficial mucosa, infiltrating inflammatory cells, deep propria glands, fundic glands and primary and metastatic cancers, suggesting that BTG1 expression pattern had cellular specificity. Compared with NNM, BTG1 expression was reduced in gastric cancer, indicating that down-regulated BTG1 expression promoted the malignant transformation of gastric epithelial cells. Although the hypoexpression of *BTG1* mRNA was found in ovarian, gastric, thyroid, hepatocellular, nasopharyngeal, esophageal, breast, and lung cancers [12–19], a genome-wide transcriptomic analysis showed an up-regulated *BTG1* expression in ovarian cancer [20]. Here, no difference in *BTG1* expression between cancer and NNM was found by RT-PCR and Western blot. This paradoxical phenomenon could be explained by BTG1 expression in stromal cells, which were excluded from the histomorphological features. Additionally, there was no association between *BTG1* methylation and mRNA expression in gastric cancers or NNM. However, we found that 5-Aza-dC treatment restored *BTG1* mRNA expression, different from the data of Kanda et al. [13]. These findings indicated that *BTG1* methylation was partially responsible for its silenced expression.

Opposite to the results from thyroid, nasopharyngeal, hepatocellular, esophageal, gastric, breast, and non-small cell lung cancers [13–19], BTG1 expression was positively linked to depth of invasion, lymphatic and venous invasion, lymph node metastasis and TNM staging of gastric cancer, which indicates that BTG1 expression might be considered as a valuable biomarker to predict aggressive behaviors of gastric cancer. Furthermore, metastatic cancers in lymph node showed a higher BTG1 expression than primary cancers, which could be explained by a similar BTG1 expression between primary and metastatic cancers, and its positive correlation with lymph node metastasis. In contrast, Chen et al. [21] reported that *BTG1* expression was higher in prostate cancer cell line LNCaP than its aggressively metastatic line, AIC4-2. Our functional experiments suggested that BTG1 overexpression might induce apoptosis and senescence, and suppress proliferation, invasion and migration. Xenograft model indicated that BTG1 overexpression might inhibit the growth and lung metastasis by inhibiting proliferation, inducing apoptosis and autophagy. In addition, ectopic BTG1 expression enhanced the chemosensitivity of gastric cancer cells to SAHA, MG132, cisplatin and paclitaxel, which was positively correlated with BTG1-induced apoptosis and lower expression of chemoresistant genes. Therefore, we speculate that BTG1 overexpression in more aggressive gastric cancers might be a reactive up-regulation and inhibit aggressive phenotypes of cancer cells in a negative feedback manner. BTG1 might be employed as a potential target for gene therapy for gastric cancer.

In agreement with Kanda data [13] about *BTG1* mRNA, a higher BTG1 expression was found in intestinal-than diffuse-type gastric carcinoma, which was supported by BTG1-induced differentiation. Moreover, BTG1 was more expressed in elder men than younger women patients, possibly due to its overexpression in intestinal-type carcinoma commonly seen in the former population [22]. Chang et al. [23] found that BTG1 was more expressed in androgen-dependent LNCaP-FGC cells and concluded that androgen might enhance BTG1 expression, supporting our result. In the present study, intestinal- and diffuse-type carcinomas showed a lower BTG1 expression than aggressive mixed-type ones [22], in consistence with the positive association between BTG1 expression and aggressive behaviors of gastric cancer. No difference in BTG1 expression detected between intestinal and diffuse components supported the notion that different components of mixed-type carcinomas might originate from common stem cells, but follow distinct histogenic pathways [22].

Reportedly, both Cyclin D1 and E1 activate CDKs and promote G_1_-S transition, which is inhibited by p21. CDC25B activates the cyclin dependent kinase cdc2 for entry into mitosis [24]. BTG1-induced G_2_/M arrest was positively linked to *Cyclin D1* overexpression, as well as *cdc2* and *p21* hypoexpression. The apoptosis-inducing effect of BTG1 might result from the *Bax* overexpression because of Bax-mediated mitochondrial voltage-dependent anion channel opening for apoptosis [25]. It was worth noting that the inhibitory effect of BTG1 on migration and invasion might be positively linked to *MMP-9* hypoexpression because MMP-9 degrades various components of the extracellular matrix and enhances the tumor invasive and metastatic potentials [26].

BTG1 expression was documented as a marker for favorable prognosis in thyroid, hepatocellular, esophageal squamous cell, breast and non-small cell lung cancers [15–19]. Here, a positive link between BTG1 expression levels and poor survival of gastric cancer patients was revealed and the significance of the relationship disappeared if stratified according to depth of invasion, indicating that the prognostic value of BTG1 expression depended upon invasive depth of the cancers. Kamalakaran et al. [27] identified CpG islands methylation of *BTG1* as a prognostic value independent of subtypes and other clinical factors of luminal breast cancers. Multivariate analysis demonstrated that venous invasion, distant metastasis and TNM staging were independent prognostic factors for overall gastric cancers. These findings indicate that BTG1 expression is an indicator for the worse prognosis of gastric cancer patients, albeit not independent of other parameters.

In summary, our study indicated that down-regulated BTG1 expression might promote the malignant transformation of gastric epithelial cells. Promoter methylation of *BTG1* partially underlies the molecular mechanisms of its down-regulated expression. BTG1 overexpression might reverse aggressive phenotypes and be used as a molecular target for gene therapy in the future.

## MATERIALS AND METHODS

### Cell lines and culture

Gastric cancer cell lines, MKN28, AGS, BGC-823, MGC-803, MNK45 and SGC-7901, KATO-III, HGC-27, GT-3 TKB and STKM-2, SCH and gastric epithelial cell line, GES-1 come from Japanese Physical and Chemical Institute, Beijing Institute for Cancer Research, Beijing, China, and Cell bank of Chinese Academy of Sciences, Shanghai, China, respectively. They were maintained in RPMI 1640, MEM, DMEM and Ham F12 medium supplemented with 10% fetal bovine serum (FBS), 100 units/ml penicillin, and 100 μg/ml streptomycin in a humidified atmosphere of 5% CO_2_ at 37°C. To demethylate the genomic DNA, cells were seeded and treated with 10 mmol/L of the DNA demethylating agent 5-aza-2′-deoxycytidine (5-Aza-dC, Sigma) for 72 h and then were harvested to extract RNA and DNA. To check the drug sensitivity, we exposed cells to cisplatin (a platinum-containing DNA crosslinker), MG132 (a proteasome inhibitor), paclitaxel (a mitotic inhibitor), and SAHA (a histone deacetylase inhibitor).

### Plasmid construction and transfection

BTG1 gene was amplified using forward primer: 5′-CCGGAATTCATGCATCCCTTCTACACC-3′ backward primer 5′-GCTCTAGAACCTGATACAGTCAT CATAT-3′, and the template cDNA from MKN28 cells. PCR products were inserted into pcDNA3.1 (Clontech, USA) between *EcoR*I and *Xba*I, which was directly sequenced. BGC-823 and MKN28 cells were transfected with pcDNA3.1-*BTG1* or pcDNA3.1 vector after seeding on dishes, selected by G418 according to the manufacturer's instructions (QIAGEN, USA) with final collection of monoclones.

### Proliferation assay

Cell counting Kit-8 (CCK-8, Japan) was employed to determine the number of viable cells. In brief, 2.5 × 10^3^ cells/well were seeded on 96-well plate and allowed to adhere. At different time points, 10 μL of CCK-8 solution was added into each well of the plate and the plates were incubated for 3 h in the incubator and measured at 450 nm.

### Cell cycle analysis

The cells were trypsinized, collected, washed by PBS and fixed in 10mL ethanol for 2 h. Then, the cells were washed by PBS and incubated with 1mL RNase (0.25 mg/mL) at 37°C for 1 h. The cells were pelleted, resuspended in propidium iodide (PI) at a concentration of 50 μg/mL and incubated at 4°C in the dark for 30 min. Finally, flow cytometry was employed to examine PI signal.

### Apoptosis assay by flow cytometry

Flow cytometry was performed with FITC-labeled annexin V and PI staining (BD Pharmingen) to detect phosphatidylserine externalization as an endpoint indicator of apoptosis according to the manufacturer's instructions.

### Wound healing assay

1.0 × 10^6^ cells /well were seeded in 6-well culture plates. After they had grown to confluence, the cell monolayer was scraped with a pipette tip to create a scratch, washed by PBS for three times and cultured in the FBS-free medium. Cells were photographed at 24 h and 48 h with the scratch area measured using Image software.

### Transwell chamber assays

For invasive assay, 2.5 × 10^5^ cells were resuspended in serum-free RPMI 1640, and seeded in the matrigel-coated insert on the top portion of the chamber (BD Bioscience, 354481). The lower compartment of the chamber contained 10% v/v FBS as a chemoattractant. After incubated at 37°C and 5% CO_2_ for 24 h, cells on the membrane were scrubbed, washed with PBS, fixed in 100% methanol and stained with Giemsa dye for the measurement. For migration assay, the procedures were the same as above excluding membrane-control insert (BD Bioscience).

### Alkaline phosphatase (ALP) activity

ALP activity was used as a marker of cellular differentiation. The cells were harvested, broken and subjected to the determination of ALP activity using Diagnostics ALP reagent (Sigma, USA). The protein content of the samples was determined by Biorad protein assay kit. ALP activity was calculated as U per μg of protein.

### β-galactosidase staining

β-galactosidase staining was performed with a senescence-associated β-galactosidase staining kit (Beyotime). Cells were washed three times with PBS and fixed with 4% paraformaldehyde for 15 min. Next, the cells were incubated overnight at 37°C in darkness with the working solution containing 0.05 mg/mL X-gal. Finally, cells were examined under a light inverted microscope (Olympus).

### Subjects

Gastric cancer (*n* = 613), adjacent non-neoplastic mucosa (NNM, *n* = 577) and lymph node with metastases (*n* = 177) were collected from surgical resection in the Affiliated Hospital of Kanagawa Cancer Center (Japan). The patients with gastric cancer were 427 men and 186 women (24∼87 years, mea*n* = 62.1 years). Intestinal and diffuse components were subjected to establishment of tissue microarray in 112 mixed-type carcinomas. Fresh gastric cancer and matched NNM (*n* = 36) were collected from the First Affiliated Hospital of Liaoning Medical University and frozen at −80°C for protein, RNA and DNA extraction. None of the patients underwent chemotherapy, radiotherapy or adjuvant treatment before surgery. They or their relatives provided written consent for use of tumor tissue for clinical research. Kanagawa Cancer Center and our University Ethical Committee approved the research protocol. We followed up the patients by consulting their case documents and by telephone.

### Pathology

All tissues were fixed in 10% neutral formalin, embedded in paraffin and sections cut at 4 μm. These sections were stained by hematoxylin-and-eosin (HE) to confirm their histological diagnosis and other microscopic characteristics. The tumor-node-metastasis (TNM) staging for each gastric cancer was evaluated according to Union Internationale Contre le Cancer system [28]. Histological architecture of gastric cancer was expressed in terms of Lauren's classification [22]. Elastic-van Gieson staining and D2–40 immunostaining were employed to characterize the venous and lymphatic invasion respectively. Furthermore, depth of invasion, lymph node and distant metastasis were determined.

### Xenograft models

Female BALB/c nude mice of 6–8 weeks were bred and used for implantation. The animals were maintained under specific pathogen-free conditions. Housing and all procedures were performed according to protocols approved by the Committee for Animal Experiments guidelines on animal welfare of Liaoning Medical University. Subcutaneous xenografts were established by injection of 1 × 10^6^ cancer cells /mouse to the axilla (*n* = 10/group). Tumor growth was then monitored for 12 days. At the end of the experiment, mice from each group was anesthetized, photographed, and sacrificed for further analysis. Tail vein assay of cancer metastasis was performed by intravenous injection of 1 × 10^6^ cancer cells (*n* = 10/group). For each tumor, measurements were made using calipers, and tumor volumes were calculated as follows: length × width × depth × 0.52. The cancer foci on lung were also counted after mandelic acid staining. The tumor and lung tissues were subsequently fixed in 4% paraformaldehyde for 24 h, and then embedded in paraffin for block preparation

### Computed tomography (CT)

GE LightSpeed CT was employed to image the tumor size and lung metastasis. Briefly, the animals were transported to and positioned in CT scanner while still fixed to their poly-styrene bed. Before and after administering a booster dose of 20 μL of contrast agent Ioversol into tail vein, we scanned 2 bed positions (separated by 40.6 mm along the longitudinal axis) to acquire the chest of the mouse. The images were acquired in step-and-shoot mode with plain CT scan condition of the x-ray source set: 2.5 mm slice thickness, 2.5 mm interval, 80 kV tube voltage, 80 μA tube current, 512 × 512 matrix, 20 cm DFOV, and PedHead SFOV.

### RT-PCR and DNA direct sequencing

Total RNA was extracted from gastric cancer cell lines or tissues using QIAGEN RNeasy mini kit (Germany). Total RNA was subjected to cDNA synthesis using AMV reverse transcriptase and random primers (Takara). Oligonucleotide primers for PCR were designed and shown in Table 4. General and real-time PCR was performed according to the protocol of Hotstart Taq polymerase (Takara) and SYBR Premix Ex Taq™ II kit (Takara) respectively. The expression level was expressed as 2^−ΔCt^, where ΔCt = Ct (gene) − Ct (GAPDH) with the control as “1” for quantitative RT-PCR. Amplicons were subjected to electrophoresis in 2% agarose gel and purified with QIAquick gel extraction kit (QIAGEN, Germany). After extraction, DNA was sequenced using a BigDye Terminator v3.1 cycle sequencing kit (Applied Biosystems). The sequence data was compared using BLAST.

**Table 4:** Primer sequences selected for real-time RT-PCR

Num	Names	Primer's sequence	Distribution	AT(°C)	Product size(bp)	Extension time(s)
1	*BTG1*	F: 5′-CAAGGGATCGGGTTACCGTTGT-3′ R: 5′-AGCCATCCTCTCCAATTCTGTAGG-3′	XM_509262.3 543-715	60	173	34
2	*CyclinD1*	F: 5′-TGCCACAGATGTGAAGTTCATT-3′ R: 5′-CAGTCCGGGTCACACTTGAT-3′	NG_000002 776-937	60	162	34
3	*cdc2*	F: 5′-GGGCACTCCCAATAA-3′ R: 5′-GATGCTAGGCTTCCTG-3′	XM_572099 631-723	60	93	34
4	*P21*	F: 5′-ACTGTCTTGTACCCTTGTGCC-3′ R: 5′-AAATCTGTCATGCTGGTCTGC-3′	XM_003950827 572-679	60	108	34
5	*Bax*	F: 5′-GATTGCCGCCGTGGAC-3′ R: 5′-GCCCCAGTTGAAGTTGC-3′	DQ926869 306-393	60	88	34
6	*mToR*	F: 5′-CGCTGTCATCCCTTTATC-3′ R: 5′-TTCTTCTCCCTGTAGTCCC-3′	NM_004958 2092-2187	60	96	34
7	*MMP9*	F: 5′-TGTACCGCTATGGTTACACT-3′ R:5′-CCTCAAAGGTTTGGAAT-3′	KJ897197.1 211-399	60	189	34
8	*Slug*	F: 5′-ATGCCTGTCATACCACAA-3′ R: 5′-GAGGAGGTGTCAGATGGA-3′	KJ892161.1 180-352	60	173	34
9	*CD147*	F: 5′-TACTCCTGCGTCTTCCTCC-3′ R:5′-TGCGAGGAACTCACGAAG-3′	KJ896510.1 318-556	60	239	34
10	*GRP78*	F: 5′-GTTCTTGCCGTTCAAGGTGG-3′ R:5′-TGGTACAGTAACAACTGCATG-3′	FJ436356 600-780	60	181	34
11	*GRP94*	F: 5′-TGACGATGAAGTTGATGTGGAT-3′ R: 5′-CATCATTCTGTTAACTTCGGCTT-3′	XM_003832567.2 245-440	60	196	34
12	*FBXW7*	F: 5′-AGATGGACCAGGAGAGTG-3′ R:5′-CTTGCATGGTTTCTTTCC-3′	XM_009448414.1 554-771	60	218	34
13	*TOP1*	F: 5′-AAAGATCGAGAACACCGG-3′ R:5′-TGTTTGGTCTTCTCCTTCT-3′	XM_004062154.1 335-456	60	122	34
14	*TOP2*	F: 5′-AAAATGAAGATGCTAAGAAAAGACT-3′ R:5′-GTACAAACCAGGAACAAAAGTGACT-3′	XM_003315476.2 226-413	60	188	34
15	*GST-π*	F: 5′-CGGGCAAGGATGACTATGTGA-3′ R: 5′-GGGCTAGGACCTCATGGATCA-3′	XM_001152516 585-746	60	162	34
16	*GAPDH*	F: 5′-CAATGACCCCTTCATTGACC-3′ R: 5′-TGGAAGATGGTGATGGGATT-3′	NM_ 002046.3 201-335	60	135	34

### Methylation-specific PCR (MSP)

Genomic DNA was extracted from cell pellets and tissues using QIAamp DNA Mini kit (QIAGEN). DNA was modified chemically with sodium metabisulphite. The bisulfite-modified DNA was amplified by using primer pairs that specifically amplify either methylated or unmethylated sequences of *BTG1*. The following methylated *BTG1*-specific primers were used: sense, 5′-GTTTTTA AGTTAAAAGGAAGGAAGTC-3′; antisense, 5′-ATATCAAAAAATATTAAAAATCACGCA-3′ (*BTG1* MSP1, −149∼−289) and sense, 5′-TTTGAG GAGTTA GTTATCGAGATTC-3′ and antisense, 5′-AAATAA ATAAAAACCGCCTAACG-3′ (*BTG1* MSP2, −517∼−645). The following un-methylated *BTG1*-specific primers were used: sense, 5′-GTTTTTAAGTTAAAAGGA AGGAAGT TGT-3′; anti-sense, 5′-ATATCAAAAA TATTAAAAATCACACA-3′ (*BTG1* USP1, −149∼−289) and sense,5′-TGA GGAGTTAGTTATTGAGATTTGG-3′ and antisense, 5′-AAATAAATAAAAACCACCTAA CACA-3′(*BTG1* USP2, −517∼−645). MSP was performed in25 μL mixtures for 40 cycles using Hot-start polymerase (Takara).

### Western blot

Tissues and cells were subjected to protein extraction by homogenization or sonication in radioimmunoprecipitation lysis assay buffer. Denatured protein was separated on SDS-polyacrylamide gel and transferred to Hybond membrane, which was then blocked overnight in 5% skim milk in TBST. For immunobloting, the membrane was incubated for 15 min with ani-BTG1 (Proteintech) or −β-actin (Sigma) antibody. Then, it was rinsed by TBST and incubated with IgG conjugated to horseradish peroxidase (DAKO, 1:1000) for 15 min. All the incubations were performed in a microwave oven to allow intermittent irradiation as recommended by Li et al. [29]. Bands were visualized with LAS 4010 (GE healthcare Life Science) by ECL-Plus detection reagents (Santa Cruz). Densitometric quantification of target proteins was performed using Scion Image software.

### Tissue microarray and immunohistochemistry

Tissue microarray was done as described previously and cut at 4 μm [22]. The immunohisto-chemical procedures were performed as described previously [30]. The primary antibodies included anti-BTG1 (Proteintech), anti-ki-67 (Abcam) and anti-LC-3B (Cell Signal) antibodies. Omission of the primary antibody was used as a negative control. As indicated in Figure 5, BTG1 protein was localized in the cytoplasm. One hundred cells were randomly selected and counted from 5 representative fields of each section blindly by two independent observers (Zheng HC and Gou WF). The expression was graded and counted as follows: 0 = negative; 1 = 1–50%; 2 = 50–74%; 3 ≥ 75%. The staining intensity score was graded as follows: 1 = weak; 2 = intermediate; and 3 = strong. The scores for BTG1 positivity and staining intensity were multiplied to obtain a final score, which determines their expression as (− = 0; + = 1–2; ++ = 3–5; +++ = 6–9).

### Terminal digoxigenin-labeled dUTP nick-end labeling (TUNEL)

Cell apoptosis was assessed using TUENL, a method that is based on the specific binding O-TdT to the 3-OH ends of DNA, ensuring the synthesis of a polydeoxynucleotide polymer. For this purpose, ApopTag Plus Peroxidase *In Situ* Apoptosis Detection Kit (Chemicon) was employed according to the recommendation. Omission of TdT enzyme was considered as a negative control.

### Statistical analysis

Statistical evaluation was performed using Spearman's correlation to analyze the rank data, Fisher's exact test to compare the different rates, and Wilcoxon test to differentiate the means. Kaplan-Meier survival plots were generated and comparisons between survival curves were made with log-rank statistics. Cox's proportional hazards model was employed for multivariate analysis. SPSS 10.0 software was applied to analyze all data and *p* < 0.05 was considered statistically significant.
